# Role of TFEB in Huntington’s Disease

**DOI:** 10.3390/biology13040238

**Published:** 2024-04-04

**Authors:** Javier Ojalvo-Pacheco, Sokhna M. S. Yakhine-Diop, José M. Fuentes, Marta Paredes-Barquero, Mireia Niso-Santano

**Affiliations:** 1 Departamento de Bioquímica y Biología Molecular y Genética, Facultad de Enfermería y Terapia Ocupacional, Universidad de Extremadura, 10003 Caceres, Spain; javierop@unex.es (J.O.-P.); smsyakhinediop@unex.es (S.M.S.Y.-D.); jfuentes@unex.es (J.M.F.); 2 Centro de Investigación Biomédica en Red en Enfermedades Neurodegenerativa, Instituto de Salud Carlos III (CIBER-CIBERNED-ISCIII), 28029 Madrid, Spain; 3 Instituto Universitario de Investigación Biosanitaria de Extremadura (INUBE), 10003 Caceres, Spain

**Keywords:** Huntington’s disease, mHTT, lysosome, autophagy, TFEB

## Abstract

**Simple Summary:**

Huntington’s disease is an inherited neurodegenerative disease caused by a mutation in the gene encoding the huntingtin protein, which leads to its accumulation and neuronal death. There are several therapeutic strategies aimed at reducing the levels of mutant huntingtin, one of which is to activate cellular degradation systems such as autophagy. The transcription factor TFEB is the key regulator of gene expression in the autophagy–lysosomal pathway. In this review, we describe how the modulation of TFEB expression in HD models affect the levels of mutant huntingtin. Further studies are needed to assess whether targeting TFEB or stimulating autophagy could be a suitable therapeutic strategy to reduce the HD phenotype.

**Abstract:**

Huntington’s disease (HD) is an autosomal dominant neurodegenerative disease caused by an expansion of the CAG trinucleotide repeat in exon 1 of the huntingtin (HTT) gene. This expansion leads to a polyglutamine (polyQ) tract at the N-terminal end of HTT, which reduces the solubility of the protein and promotes its accumulation. Inefficient clearance of mutant HTT (mHTT) by the proteasome or autophagy–lysosomal system leads to accumulation of oligomers and toxic protein aggregates in neurons, resulting in impaired proteolytic systems, transcriptional dysregulation, impaired axonal transport, mitochondrial dysfunction and cellular energy imbalance. Growing evidence suggests that the accumulation of mHTT aggregates and autophagic and/or lysosomal dysfunction are the major pathogenic mechanisms underlying HD. In this context, enhancing autophagy may be an effective therapeutic strategy to remove protein aggregates and improve cell function. Transcription factor EB (TFEB), a master transcriptional regulator of autophagy, controls the expression of genes critical for autophagosome formation, lysosomal biogenesis, lysosomal function and autophagic flux. Consequently, the induction of TFEB activity to promote intracellular clearance may be a therapeutic strategy for HD. However, while some studies have shown that overexpression of TFEB facilitates the clearance of mHTT aggregates and ameliorates the disease phenotype, others indicate such overexpression may lead to mHTT co-aggregation and worsen disease progression. Further studies are necessary to confirm whether TFEB modulation could be an effective therapeutic strategy against mHTT-mediated toxicity in different disease models.

## 1. Huntington’s Disease

Huntington’s disease (HD) is a rare, incurable neurodegenerative disorder that is genetically inherited in an autosomal dominant manner. The neuropathology of HD is characterized by a chronic and progressive accumulation of misfolded protein aggregates in the cytoplasm and nucleus, leading to the dysfunction and death of specific neurons in the brain. In fact, neurons of the striatum and cortex are particularly susceptible to this degeneration. This pathological process results in distinct abnormal movements, psychiatric issues, and cognitive deficits [1,2,3,4].

**Figure 1:**
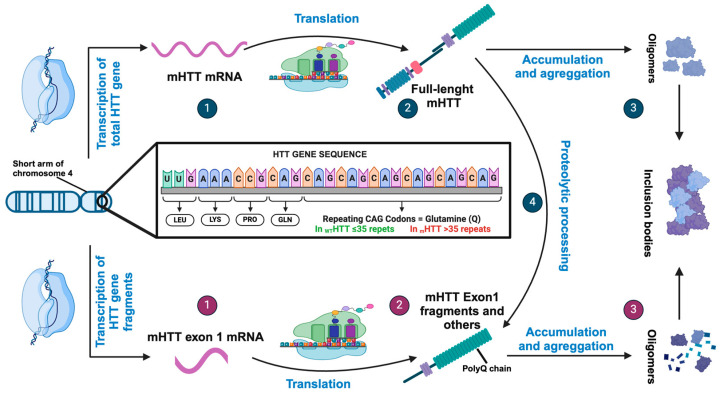
Pathogenetic mechanisms of HTT gene and HTT protein. (1) Expression of the HTT gene can generate an initial RNA transcript that is normally processed to an mRNA encoding the full-length huntingtin protein. (2) Translation produces the full-length huntingtin protein that can accumulate and aggregates (3) or can be proteolytically processed to produce a number of products, including fragments similar to HTT exon (4). However, HTT expression can be aberrantly processed to mRNA encoding only exon1 (1) if the gene contains an expanded CAG repeat and translated to HTT exon1 protein (2). These fragments containing expanded polyQ segments accumulate and aggregate (3), facilitating the formation of inclusion bodies.

Although the mechanism of pathogenesis is still unclear, the genetic mutation responsible for HD has been well established [3,4]. In 1983, HD was associated with the short arm of chromosome 4 (4p16.3) and in 1993, the gene IT15—a novel gene without known homologs—was identified. This gene contains a variable number of a cytosine-adenine-guanine (CAG) trinucleotide repeats, ranging from 6 to 180, in its first exon. IT15 encodes a protein termed huntingtin (HTT), which has a molecular weight of 348 kDa. The number of CAG repeats in HTT correlates with the age of onset and disease severity. Asymptomatic individuals have between 9 and 35 CAG repeats, with a median of 17 to 20 repeats, whereas adult-onset HD patients usually exceed 35 repeats [2,5,6,7] (Figure 1).

### HTT and mHTT

HTT protein is essential for life [7]. It is a soluble protein widely expressed in many tissues in the body but is present in higher concentrations in the brain, specifically in the striatum and cerebral cortex [5,7,8,9,10]. The function of HTT remains unknown, although it seems to play a key role in the functioning of the nervous system [8,11]. This protein contains HEAT motifs, indicating its role as a scaffolding protein [11] which allows it to interact with other proteins and a great number of cellular components. Therefore, HTT participates in numerous molecular mechanisms including those involved in vesicle transport, endosomal-lysosomal organelles, transcription, and metabolism [5,7,12,13,14,15]. In HD, there is a specific increase in the activity of the proteases. Wild-type and mutant HTT (mHTT) proteins undergo a myriad of different post-translational modifications including phosphorylation, SUMOylation, ubiquitination, acetylation, proteolytic cleavage, and palmitoylation at several sites [14]. After, they are cleaved by a variety of proteases, including several caspases, calpain and cathepsins. Cleaved N-terminal fragments with an expanded polyQ tract are translocated to the nucleus, where they are toxic, and form fibrillary aggregates or inclusion bodies, causing neuronal cell death by interfering with transcription. The soluble forms of mHTT protein are known to be ubiquitinated and immediately degraded by proteolytic systems in healthy individuals. However, when proteostasis systems fail, due to genetic variation, aging, or lifestyle, this can lead to the aggregation of mHTT, formation of inclusion bodies, and ultimately cell death [2,8,15].

Post-translational and proteolytic modifications of the HTT protein could not only impact on HTT localization and/or function but also on the function of other proteins. The amino-terminal fragments with a normal number of CAG repeats, the full-length mutant, and full-length wild-type HTT are present in the cytoplasm. In contrast, fragments with polyQ repeats are present both in the cytoplasm and nucleus, where they can accumulate [5,16]. It is not clear whether a soluble fragment of mHTT, the formation of inclusions, or both contribute to the observed toxicity. The mHTT aggregates were initially described as the toxic species in HD. However, inclusions of mutant HTT were also described as being protective, as they reduce the level of the toxic soluble mHTT protein [2].

The removal of cytosolic forms of mHTT occurs preferentially via the autophagy–lysosomal pathway, in part through HTT acetylation, whereas wild-type HTT and mHTT in the nucleus are normally degraded via the proteasome system [17]. It has been hypothesized that aggregated proteins inhibit the proteasome and thus preventing protein degradation. However, it has been shown that proteasome dysfunction is the result of simple competition for limited proteasome capacity [18].

In this paper, we will review the molecular mechanisms implicated in the neurodegenerative process characteristic of HD, with a particular focus on the deregulation of proteolytic mechanisms. Among various of them, we will discuss the role of lysosome-mediated autophagy and the role of TFEB in the progression of HD.

## 2. Alteration in HD

The accumulation of harmful proteins, such as misfolded proteins and protein aggregates, leads to cellular dysfunction and cell death, which is the main neuropathological feature of neurodegenerative diseases. To prevent this accumulation, cells maintain a protein homeostasis network known as proteostasis. Proteostasis encompasses the cellular processes that control the biosynthesis, post-translational processing, folding, trafficking, and degradation of proteins. However, as we age, these proteostasis systems become dysfunctional, contributing to the development of age-related diseases. The autophagy–lysosomal pathway, chaperone-mediated degradation, and the ubiquitin–proteasome system (UPS) are thought to be the main mechanisms for removing misfolded and abnormal proteins [10,12,19]. The intracellular accumulation of N-terminal HTT fragments and mHTT proteins in HD suggest a malfunction of the clearance systems, although not all N-terminal fragments of mHTT demonstrate selective HD-related toxicity. It is unknown which form of the expanded polyQ (misfolded, oligomeric, or aggregated) proteins represents the most toxic variants [20,21,22]. Chaperones have a direct role in modulating mHTT aggregation and toxicity by either inhibiting aggregate formation or promoting degradation via the UPS, thereby reducing the accumulation of mHTT oligomers and aggregates [14]. However, mHTT can impair ubiquitin–proteasome activity and interfere with target recognition, with each of these actions compromising autophagic clearance [2,23,24,25]. Indeed, autophagy is a catabolic process that removes dysfunctional cytoplasmic components and is able to degrade large protein complexes and aggregates [10,14,26]. Interestingly, the induction of aggrephagy, a type of macroautophagy that targets protein aggregates, facilitates the clearance of mHTT aggregates. Studies on the HD103Q mouse model (103 CAG repeats in exon 1 of the Htt gene) and neurons derived from fibroblasts of HD patients reveal the role of the FYVE (Alfy/Wdfy3) protein, a large PI3P-binding protein associated with autophagy, in the removal of mHTT aggregates [27]. The depletion of this protein results in the accumulation of mHTT and the onset of motor and neuropathological symptoms in the HD103Q mouse model [27]. Therefore, autophagy may play an important role in the elimination of mHTT. It has been proposed that HTT may participate directly in, at least, three types of selective autophagy (aggrephagy, lipophagy, and mitophagy) due to its structural similarity with mammalian target of rapamycin (mTOR), acting as a scaffolding protein for various autophagy proteins to facilitate cargo recognition [4,11,25,28,29,30]. mHTT is known to be associated with defects in autophagy. Some authors believe that mHTT abnormally activates or increases the autophagy pathway, with an unusually high formation of autophagosomes. But this is due to an impaired ability of autophagic vacuoles to recognize and fuse with lysosomes, leading to a reduced capacity of cells to degrade aggregated proteins and organelles and an accumulation of empty autophagy vesicles [2,16,31]. Others think that the defect in autophagy is not due to a failure in fusion or a reduction in proteolytic activity, but comes from an inefficient cargo loading during engulfment, which will also lead to an accumulation of “empty” vacuoles [17,32].

**Figure 2:**
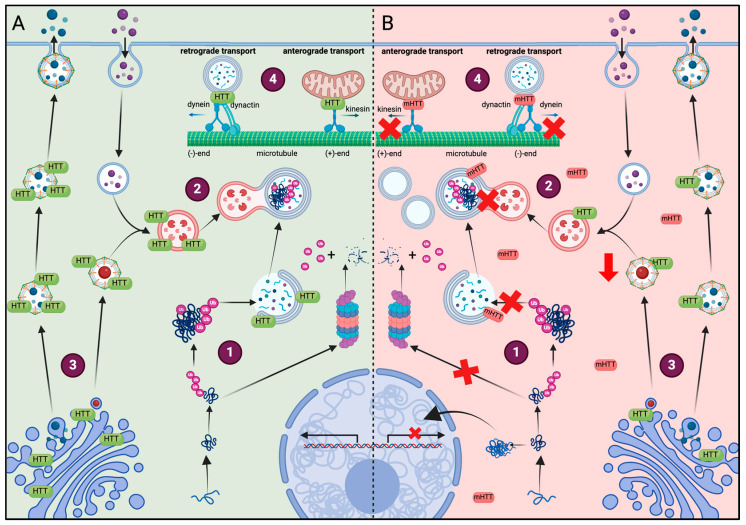
Comparison of the effect of HTT or mHTT on different cell functions. (**A**) Healthy conditions: (1) soluble fragments of HTT are prone to aggregate and are degraded by the proteasome after ubiquitination. (2) mHTT aggregates that are too large to be degraded by the proteasome are removed by autophagy. The HTT protein acts as a scaffolding protein and facilitates cargo recognition in the autophagosome. (3) HTT colocalizes with the Golgi apparatus, clathrin-coated vesicles and lysosomes, regulating their secretion and function. (4) HTT regulates the transport dynamics of mitochondria and autophagosomes by interacting with dynein, dynactin and kinesin-1. (**B**) Huntington disease: (1) due to the amount of soluble fragments of HTT, not all of them can be ubiquitinated and some form oligomers that translocate to the nucleus and interfere with the transcription of various genes. The ubiquitinated fragments accumulate in the cells because of limited proteasome capacity, leading to proteasome dysfunction. (2) mHTT alters autophagy by preventing the formation of autophagolysosomes or the recognition of mHTT aggregates, incrementing the number of empty autophagosomes. (3) The presence of mHTT in cells reduced the levels of HTT in the Golgi apparatus, reducing the secretion and formation of lysosomes. (4) mHTT disrupts mitochondrial movement and transport of autophagosomes, contributing to their accumulation.

Specifically, HTT interacts with the Golgi apparatus, exocytic vesicles and lysosomal membranes, supporting its role in the cellular trafficking and autophagy. Consequently, the presence of mHTT in cells reduces the levels in HTT in the Golgi apparatus, leading to a decrease in clathrin-dependent trafficking from Golgi to lysosomes and affecting secretion to the plasma membrane (Figure 2) [33,34,35]. Moreover, mHTT forms aggregates through its N-terminal domain, while its C-terminal domain is essential for binding to ULK1 and p62 proteins, ensuring the spatial proximity between cargo recognition and autophagy initiation components. In parallel, the N-terminal domain also participates in the activation of autophagy as mHTT aggregates sequester and inactivate mTOR [14,28], a kinase that negatively regulates autophagy. These actions support a dual role of mHTT that can both promote the initiation of autophagy and simultaneously compromises the cargo delivery to lysosomes.

As HTT acts as a scaffold protein, it also regulates the transport dynamics of several organelles by binding directly to dynein and interacting with dynactin and kinesin-1. For example, decreased HTT is known to impair mitochondrial transport because soluble mHTT fragments disrupt the formation of transport complexes and interfere with the interaction between trafficking proteins and mitochondria, impairing mitochondrial movement. Similarly, the defective autophagosome transport observed with mHTT leads to aberrant autophagosome accumulation [35,36,37,38]. In addition to affecting mitochondrial trafficking, soluble N-terminal mHTT fragments also impact various mitochondrial functions, including energy production and calcium handling [38]. Moreover, mHTT modifies the subcellular lysosomal distribution, accumulating significantly in the perinuclear regions rather than being evenly distributed throughout the cell [35] (Figure 2).

## 3. TFEB

Lysosomes are dynamic organelles essential for maintaining proper cellular homeostasis by participating in several essential cellular processes including endocytosis, autophagy, and exocytosis. Failure of lysosomal function leads, among other consequences, to the accumulation of protein aggregates [35]. Therefore, lysosomal biogenesis and activity are controlled by a variety of intracellular and extracellular signals. The biogenesis of lysosomes is highly regulated by a transcriptional network of genes known as the CLEAR network, with transcription factor EB (TFEB) acting as the master regulator [39]. TFEB belongs to the MiT/TFE family of transcription factors and serves as a central regulator of the autophagy/lysosomal-to-nucleus signaling pathway [40]. The MiT family comprises four closely related and evolutionarily conserved members: microphthalmia-associated transcription factor (MITF), transcription factor EB (TFEB), TFE3, and TFEC [41]. All members of the MiT family contain highly conserved functional domains that enable them to bind to DNA and form homodimers and heterodimers. However, outside of these regions, they differ significantly [41]. TFEB, MITF, and TFE3 contain a conserved activation domain that is crucial for their transcriptional activation [42,43]. Both TFEB and TFE3 directly bind to the CLEAR motif, which coordinates expression and regulation of diverse lysosomal functions including autophagy, lysosomal biogenesis, lysosomal exocytosis, and plasma membrane repair [44,45].

### 3.1. Regulation of TFEB Activity

**Figure 3:**
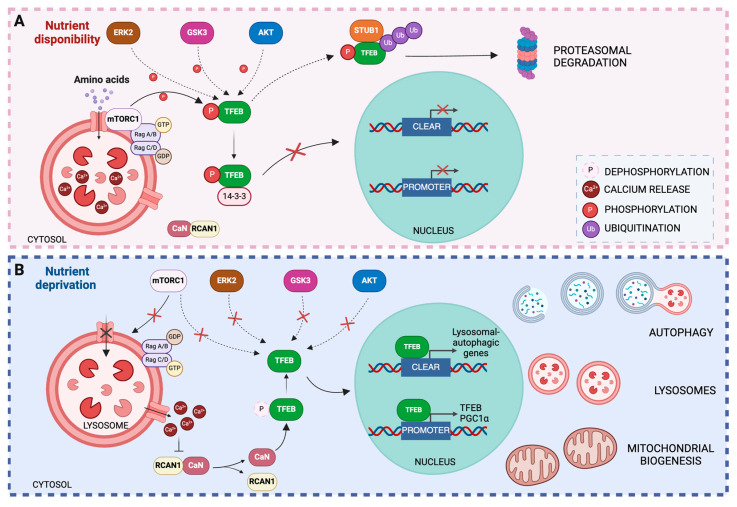
**Regulation of TFEB activity**. (**A**) Under nutrient-rich conditions, mTOR is recruited to the lysosomal membrane and activated by Rag GTPases in response to amino acids in the lysosomal lumen. mTOR activation promotes the recruitment of TFEB and its phosphorylation at serine 211. Phosphorylated TFEB is recognized by the 14-3-3 chaperone, which binds to TFEB and facilitates its sequestration in the cytosol. In addition, other protein kinases may promote TFEB phosphorylation by preventing its translocation to the nucleus and binding to the CLEAR promoter. (**B**) Under stress conditions, such as nutrient deprivation, lysosomal Rag GTPases are inactivated, resulting in the inactivation of mTORC1, which dissociates from the lysosomal surface. Ca^2+^ exits the lysosomes, increasing the cytosolic Ca^2+^ concentration and triggering the release of RCAN1 protein from calcineurin, which, once activated, dephosphorylates TFEB at Ser211, contributing to its nuclear translocation. Inside the nucleus, TFEB binds to the CLEAR motif and increases the expression of genes involved in autophagy and lysosomal biogenesis. TFEB also binds to its own promoters, thereby increasing its expression. Additionally, TFEB activates the transcription of PGC-1α, contributing to mitochondrial biogenesis.

TFEB activity is regulated by post-translational modifications, protein–protein interactions as well as its subcellular localization. The intracellular localization of TFEB is controlled by phosphorylation [46]. One of the major kinases known to phosphorylate TFEB is the mTOR kinase [47,48,49]. The activity of mTOR is regulated by various stimulus including nutrient and growth factor availability as well as stress and energy status. Under nutrient-rich conditions, the entrance of amino acids into the lysosome promotes the recruitment of mTORC1 to lysosomal membrane [49]. Here, it is activated by Rag GTPases, stimulating TFEB phosphorylation [50,51]. The phosphorylation of serine residues in the TFEB protein plays a crucial role in determining its cytosolic localization. For example, the phosphorylation of Ser211 serves as a docking site for the 14-3-3 chaperone, which retains TFEB in the cytosol and prevents its translocation to the nucleus [47]. Additionally, ERK2, AKT, and GSK3β can also phosphorylate TFEB [52,53,54]. Interestingly, STUB1, an E3-ligase, recognizes phosphorylated TFEB and targets it for proteasomal degradation [55]. Under cellular stress conditions, Rag GTPases become inactive, resulting in the inactivation of mTORC1. This causes mTORC1 to dissociate from the lysosomal surface, which triggers Ca2+ efflux from lysosomes. The elevation of cytosolic Ca2+ activates calcineurin, which dephosphorylates TFEB at Ser211, contributing to its nuclear translocation [56]. Once in the nucleus, TFEB binds to the CLEAR motif and increases the expression of genes involved in autophagy and lysosomal biogenesis. TFEB binds to its own promoters and increases their expression by autoregulatory loops [57]. TFEB is also a key regulator of lipid metabolism and mitochondrial biogenesis through the transcriptional activation of peroxisome proliferator-activated receptor-gamma co-activator-1α (PGC-1α), which is a co-activator of the nuclear receptor peroxisome proliferator-activated receptor α (PPARα) [57,58] (Figure 3).

### 3.2. TFEB in Huntington’s Disease

Dysfunction of TFEB can lead to alterations in the autophagy–lysosomal pathway, which is implicated in a variety of diseases, including lysosomal storage disorders, cancer, neurodegenerative, and metabolic diseases [59]. TFEB has been postulated as a potential therapeutic target due to its ability to regulate lysosomal activity, which is crucial for the elimination of defective proteins that accumulate in neurodegenerative diseases.

Several studies have demonstrated the role of TFEB in the clearance of aggregates in various neurodegeneration models [60,61,62,63,64,65,66,67,68]. In Alzheimer’s disease models, TFEB expression promotes the reduction of neurofibrillary tangles by activating lysosomal function [60,61]. In addition, TFEB expression in astrocytes promoted the clearance of β-amyloid in lysosomes [62,63]. In cellular and mouse models of Parkinson’s disease, genetic or pharmacological activation of TFEB promoted the activation of autophagy and reduced the levels of α-synuclein aggregation [64,65], a key hallmark of the disease. In HD, TFEB expression stimulates autophagy and lysosomal activity, leading to a decrease in mHTT levels [66,67,68] (Table 1).

One study suggests that increasing TFEB expression levels in the striatum of HD Q175/Q7 mice may be a potential therapeutic strategy for HD [66]. This transgenic mouse model expresses a mutant form of the HTT protein with 175 CAG repeats (Q175) and 7 proline-rich regions (Q7), particularly in the brain regions affected by HD, such as the striatum. TFEB expression in the striatum of HD Q175/Q7 mice stimulates autophagy and lysosomal activity, leading to a reduction in mHTT levels [66]. Consistent with these findings, elevating TFEB protein levels may have therapeutic implications in alleviating the pathological effects of mHTT accumulation in the brain. In fact, TFEB expression is significantly decreased in cultured cells and in the brains of the N171-82Q transgenic mouse model. In this model, mice express a fragment of the HTT protein with 82 glutamine repeats under the control of N-terminal HTT protein. This model displays phenotype abnormalities observed in human HD. However, it has been demonstrated that overexpressing PGC-1α, which occupies and activates TFEB promoter, thereby enhancing TFEB expression, can almost completely eliminate HTT protein aggregates in the brains of HD mice. This reduction in aggregates results in the rescue of HD neuropathology [67]. Additionally, a fully functional autophagy pathway is necessary for PGC-1α-mediated rescue of HTT protein aggregation. When TFEB is knocked down in the presence of PGC-1α overexpression, there is no appreciable reduction in HTT protein aggregation [67]. Recently, an in vitro model of HD has been used to further demonstrate the involvement of TFEB function in HTT aggregates. In this in vitro model, fibroblasts from HD patients are reprogrammed into medium spiny neurons (HD-MSNs), a type of neuron significantly affected in HD. This model replicates the late-onset pathology of HD and preserves the epigenetic markers from the fibroblasts. Interestingly, the TFEB level is dysregulated in HD-MSNs; however, it has been demonstrated that reducing the interaction between the regulator of calcineurin (RCAN1) and calcineurin (CaN) promotes the nuclear localization of TFEB and the clearance of HTT inclusion bodies as well as enhances neuronal survival of HD-MSNs [68]. All these findings suggest that TFEB activation through different mechanisms reduce mHTT aggregation levels in HD models in vitro and in vivo. However, it is important to note that the level and duration of autophagy activation by TFEB must be strictly controlled, as an excessive expression of TFEB can disrupt cellular homeostasis. A recent study investigated how different methods and timing of autophagy activation affect the efficacy of autophagy-activating treatment in vivo. Surprisingly, they found that co-injection of AAV5-TFEB (human TFEB) vector together with AAV-mHTT (exon 1 of human mHTT) into the mouse striatum did not reduce the aggregation of mHTT [69]. These results suggest that the activation of autophagy by TFEB is not sufficient to effectively reduce mHTT accumulation, because the degradation process does not occur due to cargo recognition problems, resulting in the accumulation of empty autophagosomes [69]. In line with these findings, Yang et al. have demonstrated the unexpected co-aggregation of TFEB with mHTT aggregates and its possible implications for HD pathogenesis and therapeutic strategies [70]. The study was conducted in HD cell models (Hela and mouse neuroblastoma N2a cells) and in the HdhQ140 knock-in mouse model. The HdhQ140 model carries 140 CAG repeats in the mouse huntingtin gene (Hdh) and exhibits features of HD such as the formation of HTT aggregates, motor dysfunction, and neuronal degeneration. The study observed co-aggregation of endogenous TFEB with mHTT in N2a cells, potentially mediated by a prion-like domain (PrLD) in TFEB. Furthermore, immunofluorescence analysis of HdhQ140 mouse brains revealed co-localization of TFEB and mHTT aggregates, reinforcing the relevance of the co-aggregation phenomenon in the context of HD pathogenesis [70]. In conclusion, this study presents evidence for co-aggregation of TFEB with mHTT in both in vitro and in vivo, highlighting the potential importance of this interaction in the context of HD pathogenesis. The identification of PrLD in TFEB as a mediator of co-aggregation opens new avenues for the investigation of the molecular mechanisms underlying HD and for the development of targeted HD therapies.

## 4. Conclusions and Future Perspectives

Currently, the major focus is on developing new therapeutic strategies that target the pathogenesis of HD, specifically, mHTT aggregates. These approaches aim to reduce mHTT levels and mitigate the various pathogenic effects. In animal models of HD, reducing mHTT levels has improved the disease phenotype and reversed neuropathology, confirming the importance of mHTT reduction as a therapeutic target. This review summarizes the major findings on the role of the TFEB protein in the clearance of mHTT aggregates. The autophagic–lysosomal pathway is important for clearance of mHTT protein aggregates, and this process is altered in both animal models of the disease and in tissue from HD patients. Several studies demonstrated that activation of the autophagic–lysosomal pathway via TFEB protein could be a promising therapeutic strategy because of its ability to regulate lysosomal activity and coordinate a transcriptional program that controls the autophagic degradative pathway. However, it is important to note that the level and duration of autophagy activation by TFEB must be strictly controlled. A recent study has shown that overexpression of TFEB can exacerbate the disease by increasing the formation of mHTT aggregates. The researchers observed that the aggregation of TFEB and mHTT is mediated by the PrLD domain at the N-terminal end of TFEB, a domain that, interestingly, is not found on TFE3, another MiT family transcription factor that shares functions with TFEB to regulate autophagy. Future studies with this transcription factor are needed as it may serve as an alternative drug target for HD.

The effective reduction in mHTT levels by the autophagy–lysosomal pathway is a therapeutic challenge because of the complexity of the autophagic process, especially when this process is altered in the more advanced stages of the disease. Thus, promoting the activation of autophagy in advanced stages of the disease does not lead to the clearance of mHTT aggregates, but rather to their accumulation.

In recent years, protein degradation strategies have emerged that target proteins for elimination by different cellular degradation systems. In contrast to proteasome-targeted protein degradation, which is ineffective at removing large proteins or aggregates, targeted degradation strategies based on the autophagy–lysosomal pathway, such as LYTAC, AbTAC, ATTEC, AUTAC, and AUTOTAC, have the potential to remove protein aggregates and damaged organelles. In the context of HD, a study has identified a series of small molecules that can bind to the LC3 protein and the pathogenic mHTT protein [71]. These molecules appear to recognize the conformation of the extended polyQ stretch in the mutant protein and distinguish it from the normal protein. This provides a new strategy for the treatment of HD, and it would be interesting to determine whether it could be used for other diseases caused by polyQ expansions. In addition, long-term preclinical efficacy and safety studies will be required for the therapeutic development of these compounds.

## Figures and Tables

**Table 1:** TFEB studies in Huntington’s disease.

Methods of Targeting TFEB	Model	Phenotype	Study
TFEB overexpression	Striatum of Q175/Q7 HD mice	Clearance of polyQ-HTT	[66]
TFEB expression is promoted by inducing PGC-1α expression	N171-82Q transgenic mice	Reduction of protein HTT aggregation	[67]
Enhanced calcineurin activity promotes nuclear localization of TFEB	Patient-derived striatal medium spiny neurons	Clearance of HTT inclusion bodies and neuronal survival	[68]
Overexpression of human TFEB	AAV-based model of HD	No change in the amount of mHTT aggregates	[69]
TFEB overexpression	HdhQ140 mice	Co-aggregation of TFEB and mHTT	[70]

## Data Availability

Not applicable.
